# Zebrafish Whole Mount High-Resolution Double Fluorescent *In Situ* Hybridization

**DOI:** 10.3791/1229

**Published:** 2009-03-25

**Authors:** Tim Brend, Scott A. Holley

**Affiliations:** Department of Molecular, Cellular and Developmental Biology, Yale University

## Abstract

Whole mount *in situ* hybridization is one of the most widely used techniques in developmental biology.  Here, we present a high-resolution double fluorescent *in situ* hybridization protocol for analyzing the precise expression pattern of a single gene and for determining the overlap of the expression domains of two genes.  The protocol is a modified version of the standard *in situ* hybridization using alkaline phosphatase and substrates such as NBT/BCIP and Fast Red ^1,2^. This protocol utilizes standard digoxygenin and fluorescein labeled probes along with tyramide signal amplification (TSA) ^3^.  The commercially available TSA kits allow flexible experimental design as fluorescence emission from green to far-red can be used in combination with various nuclear stains, such as propidium iodide, or fluorescence immunohistochemistry for proteins.  TSA produces a reactive fluorescent substrate that quickly covalently binds to moieties, typically tyrosine residues, in the immediate vicinity of the labeled antisense riboprobe. The resulting staining patterns are high resolution in that subcellular localization of the mRNA can be observed using laser scanning confocal microscopy ^3,4^.  One can observe nascent transcripts at the chromosomal loci, distinguish nuclear and cytoplasmic staining and visualize other patterns such as cortical localization of mRNA.  Studies in *Drosophila* indicate that roughly 70% of mRNAs exhibit specific patterns of subcellular localization that frequently correlate with the function of the encoded protein ^5^.  When combined with computer-aided reconstruction of 3D confocal datasets, our protocol allows the detailed analysis of mRNA distribution with sub-cellular resolution in whole vertebrate embryos.

**Figure:**
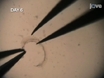


## Protocol

### 1. FIXATION

Fix embryos overnight at 4°C with 4% paraformaldehyde (PFA) in PBS.
Remove fix and wash 2 x PBS, 5 minutes each at room temperature (RT).
Manually dechorionate embryos in PBS in a glass depression plate using watchmaker forceps. After dechorionation, embryos are transferred using a fire-polished glass Pasteur pipette as they may stick to polypropylene pipettes.
Transfer embryos through a series of 25%, 50% and 75% methanol in PBS for 5 minutes each.
Replace liquid with 100% methanol, incubate 5 minutes and then replace with fresh methanol.
Place embryos at -20°C for a minimum of one hour.  (We typically incubate the embryos overnight.  Standard in situ hybridization will work on embryos stored for more than a year, but we have not examined whether the high-resolution of the fluorescent in situ hybridization is lost by prolonged storage.)
Wash embryos for 5 minutes each in 75%, 50%, 25% methanol in PBST at RT.  Wash twice for 5 minutes each in PBST at RT.
Fix again for 20 minutes in 4% PFA in PBS at RT.
Wash twice for 5 minutes each in PBST at RT.

NOTE REGARDING PFA: We store 4% PFA in aliquots at –20ºC (see table of reagents).  For the initial fixation, we only use PFA that has never been previously thawed.  For subsequent fixations, we often use PFA that has been previously thawed.  The initial fixation appears to be critical for successful staining with this protocol.

### 2. PROTEINASE AND POSTFIXATION

Digest with proteinase K (5μg/ml in PBST) at RT for 3 to 12 minutes to permeabilize the embryos.  (The incubation time depends on the age of the embryos as younger stages are more sensitive.  It also depends on the batch of enzyme.)  For somitogenesis stage embryos, we typically permeabilize for 3-4 minutes.  During this incubation, we lay the microcentrifuge tube on its side.
Rinse briefly in PBST and wash once for 5 minutes in PBST.
Fix again for 20 minutes in 4% PFA in PBS at RT.
Wash twice, for 5 minutes each, in PBST at RT.

### 3. PREHYBRIDIZATION

Incubate the embryos for 5 minutes at 65º C in HYB-.
Prehybridize at 65º C for at least 1 hour in HYB+.

### 4. HYBRIDIZATION

Remove all but 50 μl of the preHYB, but make sure to keep the embryos completely submerged.
Add 1-2 μl of each riboprobe (digoxygenin- and fluorescein-labeled riboprobes) to the embryos and mix by gently flicking the tube.  The amount of probe is typically 1-2µl from a 20µl probe synthesis reaction.
Given that fluorescein is light-sensitive, the tubes should be wrapped in aluminum foil or otherwise exposed to minimal light from this point forward.
Incubate the embryos overnight at 65ºC.

### 5. PROBE REMOVAL

Note that the solutions from this point forward lack detergent.  Elimination of detergent appears to help the staining reactions but does cause the embryos to become rather sticky.

Remove the riboprobe.
Wash 2 x 30 minutes at 65ºC in 50% formamide/2xSSC.
Wash for 15 minutes at 65ºC in 2 x SSC.
Wash for 30 minutes 65ºC in 0.2 x SSC.

### 6. ANTI-FLUORESCEIN ANTIBODY INCUBATION

Block for at least 1 hour at RT in 500μl of a solution of 1x maleic acid buffer plus 2% blocking reagent (see table of reagents).
Add the anti-Fluorescein-POD antibody, as supplied by Roche, at a 1:500 dilution in the blocking solution.
Incubate overnight at 4ºC.  During this incubation, we lay the microcentrifuge tube on its side.
Wash 4 x 20 minutes in 1x maleic acid buffer.  Wash twice for 5 minutes each in PBS.

### 7. DETECTION OF FLUORESCEIN-LABELLED PROBE

Incubate 30-60 minutes in TSA Plus Fluorescein Solution.  (Spin down TSA substrate before making staining solution. For the reaction, dilute tyramide reagent 1:50 in Perkin Elmer amplification diluent buffer.)  During this incubation, we lay the microcentrifuge tube on its side.  Reaction time must be determined empirically for each probe.  Unfortunately, the staining reaction cannot be visually monitored as the substrate is fluorescent, and one will see ubiquitous green fluorescence throughout the staining reaction.
Wash for 10 minutes each in 30%, 50%, 75% and 100% methanol in PBS.
Incubate in a solution of 1% H_2_0_2_ in methanol for 30 minutes to inactivate the first peroxidase.
Wash 10 minutes each in 75%, 50% and 30% methanol in PBS. Then wash twice for 10 minutes each in PBS.  It is important that all of the methanol be removed.

NOTES ON THE TYRAMIDE SIGNAL AMPLIFICATION: We have been using the Perkin Elmer TSA Kits.  We find that the Alexa-Tyramide substrates stain well using the Perkin Elmer amplification diluent buffer, but we have not had success using the staining buffer provided with the Invitrogen/Molecular Probes Kits.  Lastly, we have found that Cy5 fluorescence is eliminated by subsequent Methanol/H_2_O_2_ treatment while fluorescein and Alexa-647 are unaffected.  Cy3 may also be adversely affected by the Methanol/H_2_O_2_ treatment as it is structurally related to Cy5.  For this reason, Cy3 and Cy5 TSA reactions should only be used for the second staining reaction in a double fluorescent in situ protocol.

### 8. ANTI-Digoxygenin ANTIBODY INCUBATION

Block the embryos again for at least 1 hour at RT in a solution of 1x maleic acid buffer plus 2% blocking reagent.
Add the anti-DIG POD antibody as supplied by Roche at a 1:1000 dilution in above blocking solution
Incubate overnight at 4ºC.  During this incubation, we lay the microcentrifuge tube on its side.
Wash 4 x 20 minutes in 1x maleic acid buffer.  Wash twice for 5 minutes each in PBS.

### 9. DETECTION OF Digoxygenin-LABELLED PROBE

Incubate 30-60 minutes in TSA Plus Cy5 Solution (Spin down TSA substrate before making staining solution. For the reaction, dilute tyramide reagent 1:50 in amplification diluent buffer)  During this incubation, we lay the microcentrifuge tube on its side.  Reaction time must be determined empirically for each probe.
Wash three times for 10 minutes each in PBST.

### 10. PROPIDIUM IODIDE STAINING

Wash twice for 5 minutes each in 2 x SSC.
Incubate embryos for 30 minutes at 37°C in 50 μl 2 x SSCT with 10 μl RNAse (for a final concentration of 100μg/ml).
Wash six times for 3 minutes each in 2 x SSC at RT.
Stain embryos for 8 minutes in a solution of 330µg/ml propidium iodide in 2 x SSC.
Wash six times for 3 minutes each in 2 x SSC at RT.
Fix for 20 minutes in 4% PFA in PBS at RT.
Wash twice, for 5 minutes each, in PBST at RT.

### 11. MOUNTING

Incubate the embryos for 10 minutes each in 25% and 50% glycerol in PBST. Clear overnight in 75% glycerol at 4ºC.
We dissect and deyolk the embryos and flat mount them on a microscope slide.  The yolk can produce significant background fluorescence.  Dissection is much easier after the embryos have cleared ovenight in gylcerol.

#### SOLUTIONS:

**Table:** 

PBST	PBS plus 0.1% Tween	
SSCT	SSC plus 0.1% Tween	
HYB-	50% formamide5xSSC0.1% Tween-20	
HYB+	HYB-5mg/ml torula (yeast) RNA50μg/ml heparin	The torula RNA is prepared by proteinase K digestion of RNA with subsequent phenol-, phenol-chloroform-, and chloroform-extraction.The RNA is precipitated and dissolved in DEPC-treated water.
1 x maleic acid buffer	150mM maleic acid, 100mM NaCl (pH 7.5)	


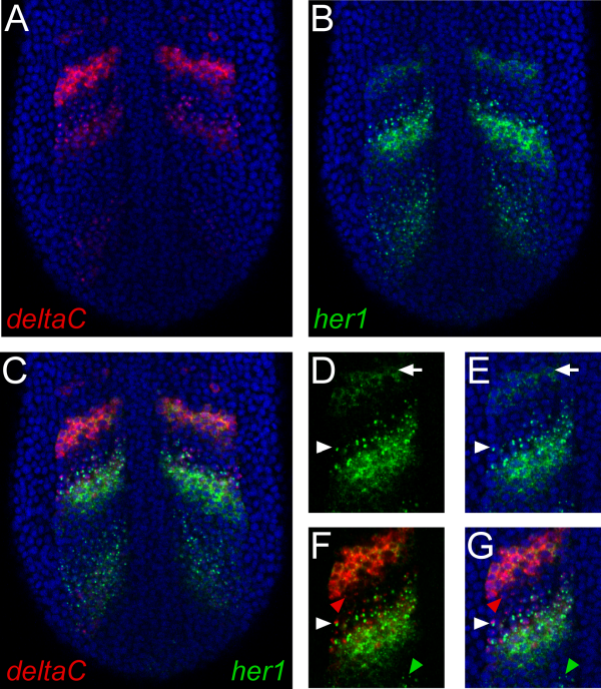


**Figure 1.** Representative results of whole mount double fluorescent in situ hybridization. (A-C). Images show a single confocal section through the posterior region of a zebrafish embryo at the ten-somite stage. The view is dorsal with anterior to the top. Nuclei stained with propidium iodide are colored blue. (A) *deltaC* mRNA was detected using a digoxygenin-labeled riboprobe and TSA-Cy5 reagent. (B) mRNA transcribed from the *her1* gene was detected with a fluorescein-labeled riboprobe and TSA-fluorescein reagent. (C) Merging the green and red channels unambiguously identifies regions of distinct or overlapping gene expression of the two genes. (D, E) Detail of *her1* expression demonstrating the high resolution of this procedure. Subcellular localization of mRNA can be clearly discerned. Arrowheads indicate a cell exhibiting active transcription, revealed by dots of staining in the nucleus. Arrows indicate a cell showing cytoplasmic localization of mRNA. (F, G) Double staining for *her1* and *deltaC* reveals cells that are transcribing both genes (white arrowhead), or either gene separately (red and green arrowheads).

## Discussion

The protocol presented here works well with probes that give a clean strong signal after staining for 30-45 minutes in a typical alkaline phosphatase-mediated reaction.  Prior to performing the fluorescent in situ hybridization protocol, we always test our probes using the standard non-fluorescent protocol (provided in supplemental material along with the probe synthesis protocol).  We have had less success with the fluorescent in situ hybridization protocol when using weaker probes, but it may be possible in some cases.  We have successfully combined the fluorescent in situ hybridization protocol with immunolocalization of β-catenin ^3^.  When performing this variation, we do the HYB-, HYB+ and hybridization incubations at 55°C instead of 65°C as the epitopes appear to be preserved better at the lower temperature.  The immunolocalization incubations are performed after the TSA reactions.

Since fluorescein-labeled probes are generally weaker than a digoxygenin-labeled probe for the same gene, we usually visualize the fluorescein-labeled probe first.  One instance where one might want to visualize the digoxygenin probe first is if one of the two genes is expressed at a low level.  In this case, one makes a digoxygenin-riboprobe for the weak gene and stains for it first to maximize the signal to noise.  Note that if you visualize the fluorescein probe second, you should not visualize the digoxygenin probe with fluorescein TSA as the anti-fluorescein antibody will recognize the TSA stain as well as the fluorescein-labeled riboprobe.
